# End-of-Life Decision-Making in Intensive Care Ten Years after a Law on Advance Directives in Germany

**DOI:** 10.3390/medicina57090930

**Published:** 2021-09-04

**Authors:** Jan A. Graw, Fanny Marsch, Claudia D. Spies, Roland C. E. Francis

**Affiliations:** 1 Department of Anaesthesiology and Operative Intensive Care Medicine (CCM, CVK) Charité—Universitätsmedizin Berlin, Augustenburger Platz 1, 13353 Berlin, Germany; fanny.marsch@charite.de (F.M.); Claudia.spies@charite.de (C.D.S.); roland.francis@charite.de (R.C.E.F.); 2 Berlin Institute of Health (BIH), Charitéplatz 1, 10117 Berlin, Germany

**Keywords:** end-of-life decision, advance directive, intensive care unit, shared decision-making

## Abstract

*Background and Objectives*: Mortality on Intensive Care Units (ICUs) is high and death frequently occurs after decisions to limit life-sustaining therapies. An advance directive is a tool meant to preserve patient autonomy by guiding anticipated future treatment decisions once decision-making capacity is lost. Since September 2009, advance directives are legally binding for the caregiver team and the patients’ surrogate decision-maker in Germany. The change in frequencies of end-of-life decisions (EOLDs) and completed advance directives among deceased ICU patients ten years after the enactment of a law on advance directives in Germany is unknown. *Materials and Methods*: Retrospective analysis on all deceased patients of surgical ICUs of a German university medical center from 08/2008 to 09/2009 and from 01/2019 to 09/2019. Frequency of EOLDs and advance directives and the process of EOLDs were compared between patients admitted before and after the change in legislation. (No. of ethical approval EA2/308/20) *Results:* Significantly more EOLDs occurred in the 2019 cohort compared to the 2009 cohort (85.8% vs. 70.7% of deceased patients, *p* = 0.006). The number of patients possessing an advance directive to express a living or therapeutic will was higher in the 2019 cohort compared to the 2009 cohort (26.4% vs. 8.9%; difference: 17.5%, *p* < 0.001). Participation of the patients’ family in the EOLD process (74.7% vs. 60.9%; difference: 13.8%, *p* = 0.048) and the frequency of documentation of EOLD-relevant information (50.0% vs. 18.7%; difference: 31.3%, *p* < 0.001) increased from 2009 to 2019. *Discussion*: During a ten-year period from 2009 to 2019, the frequency of EOLDs and the completion rate of advance directives have increased considerably. In addition, EOLD-associated communication and documentation have further improved.

## 1. Introduction

Organ support and replacement technology on the Intensive Care Unit (ICU) has converted death from a sudden and often unexpected event to a process which is dependent of the level of provision of care and organ support [1]. Consequently, patients often die after individual decisions to limit life-sustaining ICU therapy have been made [2,3]. These so-called end-of-life decisions (EOLD) differentially include measures to withhold or withdraw ICU therapeutic approaches such as cardio-pulmonary resuscitation, endotracheal intubation, mechanical ventilation, renal replacement therapy, catecholamine infusions, surgery, antimicrobial therapy, and blood product transfusions. In the recent decade, an increase in the occurrence of EOLDs was recognized across European ICUs [3].

EOLDs are based on the principle of shared decision-making between the caregiver team and the patient or his surrogate decision maker [4]. They show a regional variety due to their association with culture, religion, and different laws and healthcare systems [3]. Due to disease severity, patients on the ICU frequently lose their autonomy and self-determined decision-making capacity and cannot participate in the EOLD process [4,5,6]. With a written advance directive, patients can document their personal will, values, and preferences, including the definition of a personal surrogate decision maker in advance of an anticipated situation when decision-making capacity is lost. Furthermore, an advance directive can guide anticipated future treatment decisions.

In recent years, many countries have started to support and guide the use of advance directives by new laws and regulations [3,7,8]. Advance directives were legally approved in Germany on 1 September 2009 [8]. Since then, advance directives are legally binding for the caregiver team and the patients´ surrogate decision makers in case the anticipated decisions match with the current health status of the patient.

Although advance directives have emerged in public, only a few studies have reported the prevalence in Germany since 2009. In addition, the frequency of completed advance directives might differ among different medical subspecialties and data about patients on the ICU are poor [9].

The aim of the current study was to determine the changes in the frequency of EOLDs and the presence of completed advance directives among deceased patients in an ICU of a German tertiary care university hospital a decade after enacting a new law on advance directives in Germany.

## 2. Methods

The Medical Ethics Committee of Charité—Universitätsmedizin Berlin approved this study (number of ethical approval: EA2/308/20).

### 2.1. Setting

This is a retrospective cohort study of deceased patients who were admitted to an ICU of the Department of Anesthesiology and Intensive Care Medicine at Charité—Universitätsmedizin Berlin with in-house consultant coverage 24 h per day, 7 days a week. In addition, there is continuous coverage of the ICU by two to six residents. Daily rounds involve consultants with a board certification in intensive care medicine and at least once a day, there is a ward round involving an attending specialist surgeon from each specialty admitting patients to the ICU.

### 2.2. Patients

The study includes all consecutively admitted ICU patients who died between 1 August 2008 and 1 September 2009 on a 22-bed ICU (2009 cohort, *n* = 123) and between 1 January and 1 September 2019 on a complementary 41-bed ICU (2019 cohort, *n* = 145). Because admissions in 2019 included neurological and neurosurgical patients (*n* = 39), these patients were excluded from further analysis to reduce baseline imbalance between study cohorts. An EOLD, a do-not-resuscitate (DNR) order, and an order to withhold and/or withdraw life support (WH/WDLS) were defined as described previously [4]. All data of the 2009 cohort were previously published and are now compared to the new cohort of 2019 [4].

### 2.3. Data Sources

All data required for this study were extracted from the electronic patient data management systems of the hospital as described previously [4]. The version of the electronic patient data management system (PDMS) (Copra System, Sabachswalden, Germany) was the same for both study periods. The acute physiology and chronic health evaluation II (APACHE II) and the simplified acute physiology score II (SAPS II) were calculated automatically by the PDMS. Documentation of progress notes and limitations of therapy after EOLD conferences were documented together with the time and the participants. Patients received an EOLD only when every participant of the EOLD conference consented to the decision and the patient-individual regulations.

### 2.4. Statistical Analysis

Results are expressed as arithmetic mean ± standard deviation (SD) for continuous variables and frequencies (%) for categorical variables, respectively. Due to the sample sizes and the skewness of distributions, only nonparametric (exact) tests were applied. Differences between groups were tested by the nonparametric (exact) Wilcoxon–Mann–Whitney test for independent groups. Frequencies were tested by the (exact) Chi-square-test. A two-tailed *p*-value <0.05 was considered statistically significant. All tests were conducted in the area of exploratory data analysis. Therefore, no adjustments for multiple testing have been made. All numerical calculations were performed with statistical software package SPSS^®^ Version 27 (IBM, Armonk, North Castle, NY, USA).

## 3. Results

Table 1 shows epidemiologic data including ICU severity scores and ICU length of stay (LOS) of all 229 deceased patients that were included in the analysis. Most admissions to the ICU (58.1%) occurred as emergency admissions and 52 admissions (22.7%) were planned admissions. Compared with patients included in the 2009 cohort, patients in the 2019 cohort did not differ in median age, sex, urgency of admission, disease severity measured by ICU severity scores APACHE II and SAPS2, and in ICU-LOS (Table 1). Furthermore, with the exception of the frequency of heart failure NYHA IV, patients in the 2009 cohort compared to patients in the 2019 cohort did not differ in baseline comorbidities (Table 2). The relative number of deceased patients that were admitted after cardiac surgery was greater in the 2009 cohort compared to the 2019 cohort (2009: 57.7% vs. 2019: 34.9%; difference: 22.8%, *p* < 0.001).

Significantly more limitations of ICU therapy occurred in the 2019 cohort compared to the 2009 cohort (2019: 85.8% vs. 2009: 70.7%; difference: 15.1%, *p* = 0.006). The frequency of patients possessing an advance directive or a precautionary power of attorney was approximately three times higher in the 2019 cohort compared to the 2009 cohort of deceased patients (Table 3). The number of patients with a legal guardian during their ICU stay showed a tendency for a decrease in the 2019 cohort compared to the 2009 cohort (Table 3). Furthermore, the frequency of documentation of the EOLD-relevant information about patient-sided advance directives in the special section of the PDMS increased significantly from 18.7% in the 2009 cohort to 50.0% in the 2019 cohort (difference: 31.3%, *p* < 0.001). Taken together, these data suggest that besides an increase in the frequency of EOLDs, the number of patients with an advance directive and the efforts of appropriate documentation of EOLD-relevant information have significantly increased within the ten-year period from 2009 to 2019.

Complementary data were derived when the subgroup of deceased patients with a preceding EOLD was analyzed (Table 4). The frequency of patients possessing an advance directive or a precautionary power of attorney was higher in the 2019 cohort compared to the 2009 cohort, while the number of patients with a legal guardian during their ICU stay showed a tendency for a decrease in the 2019 cohort compared to the 2009 cohort (Table 4). Similarly, the frequency of documentation of EOLD-relevant information on advance directives in the special section of the PDMS increased significantly from 26.4% in the 2009 cohort to 54.9% in the 2019 cohort (difference: 28.5%, *p* < 0.001).

To assess the EOLD process, communication about the EOLD with the patient or the patient´s family, the timing of EOLD and death, and the frequency of step-wise escalations from a DNR order to a WH/WDLS order were analyzed (Table 4). While patient participation and patient information in EOLDs remained low after the ten-year period from 2009 to 2019, participation of the patient´s family or surrogate decision maker in the EOLD process showed a significant increase in the 2019 cohort compared to the 2009 cohort (2019: 74.7% vs. 2009: 60.9%, difference: 13.8%, *p* = 0.048, Table 4). In addition, information on the patient´s family or surrogate decision maker occurred more often in the 2019 cohort compared to the 2009 cohort of deceased patients with an EOLD (2019: 96.7% vs. 2009: 88.5%, difference: 8.2%, *p* = 0.045, Table 4). The mean number of days from ICU admission to the first EOLD and the time from the first EOLD to death did not differ between the 2009 and the 2019 cohort of deceased patients with an EOLD (Table 4). Taken together, these data suggest that EOLD communication with the patients´ next of kin has further improved during the ten-year period from 2009 to 2019, while the time required for the EOLD process to evolve remained stable.

## 4. Discussion

Among patients who had died on the ICU in 2019 compared to a complementary cohort of 2009, a significant increase in EOLDs preceding death was noted. Furthermore, the number of patients with an advance directive, the frequency of EOLD-communication with the patients´ next of kin, and efforts of appropriate documentation of EOLD-relevant information by the caregiver team have significantly increased within the ten-year period from 2009 to 2019.

The increase in ICU patients who died after an EOLD is concordant to a trend recognized for many other ICUs across Europe [3]. The large international prospective multicenter Ethicus-2 study revealed that in the years 2015 and 2016, 89.7% of all deaths on European ICUs were preceded by a decision to limit life-prolonging therapies [3]. Sprung and colleagues attributed multifactorial reasons for this progress in EOLD-making [3]. Besides new laws and governmental regulations over the recent decades, the improvement and growing integration of palliative care into intensive care medicine is held accountable for this development [7,8,10,11,12]. Furthermore, improved knowledge and consideration of the long-term outcomes of ICU therapies and the implications on the prognosis of specific disease conditions have considerably increased over the recent decade and might impact on the shared decision-making process [13,14].

So far, there are few data on the prevalence of completed advance directives among ICU patients in Germany. For the period from August 2008 to September 2009, we had already reported that 8.9% of the deceased patients of the observed cohort had completed an advance directive [4]. This number is concordant with results from surveys conducted during the respective period in Germany, including the first year after enacting the new law on advance directives [4,15,16,17,18]. Results from a survey of 998 German ICU patients in 2013 and 2014 demonstrated that 51.3% of patients claimed that they had completed a form of advance directive prior to ICU admission. However, only in 11.8% of the cases were advance directives found in the patients´ case files [9]. Furthermore, in the respective study, only ICU survivors that were fully orientated and legally competent were interviewed just before discharge to a normal ward [9]. In the current study, only deceased patients were included. In addition, most patients in the 2019 cohort, similar to the 2009 cohort, were neither informed about nor they participating in their EOLD and most of the patients were unplanned or emergency admissions to the ICU. Therefore, disease severity was probably too high at the time of the EOLD to allow participation of the patients in the EOLD process.

In association with an advance directive, in Germany often a so-called precautionary power of attorney is completed, a document in which a substitute decision maker is defined for medical decisions once decision-making capacity is lost [19]. In the current study, we demonstrated that the number of precautionary powers of attorney increased in parallel with the increasing prevalence of advance directives. Although completion rates of advance directives differ among patient subgroups and medical disciplines, recent data indicate an increasing number of patients to complete an advance directive in Germany [9,15,20,21]. Because all studies are descriptive in nature, it is unclear whether and to what extent factors like a new law or increased awareness in the media have contributed to this trend. Patient-sided factors that are robustly associated with advance directive completion rates irrespective of the medical specialty include patient age and the presence of, or experiences with, a life-threatening disease [9,17,22,23,24]. An international telephone survey suggested that interest in completing an advance directive and to preserve autonomous decision-making capacity was highest in Germany compared to any other surveyed European population, potentially because family ties are not as intense in Germany compared to many other European countries [25,26]. Considering the increasing number of completed advance directives in Germany, to date it remains unclear to what extent an advance directive impacts the decision-making process of EOLDs. Therefore, future research should focus on the relevance and importance of advance directives in the context of the corresponding EOLD.

In our previous study, we reported a significant increase (24.9%) in the EOLD-associated documentation of advanced directives in the special section of the patient chart in the PDMS after enacting the new law on advance directives in 2009 [4]. In the current study, we observed an additional increase to a documentation rate of 50% in the 2019 cohort. Although this indicates that documentation of EOLD-associated factors has become a standardized procedure, it is unclear why this documentation occurs only for half of the patients that died on the ICU. Moreover, in 2010, the German Interdisciplinary Association for Intensive and Emergency Medicine (DIVI) officially determined documentation of family conferences as one of ten indicators to measure the overall quality of an ICU and their processes [27].

The current study is limited by its retrospective and single center design. In addition, due to restructuring of intensive care therapy at Charité—Universitätsmedizin Berlin within the ten-year period from 2009 to 2019 and a concomitant increase of bed capacity, there was an imbalance of patients from different medical specialties primarily admitting to their respective ICU. Exclusion of neurological and neurosurgical patients reduced the imbalance, while still leaving fewer admissions from surgical subspecialties, including fewer admissions after cardiac surgery in the 2019 cohort compared to the 2009 cohort. However, the main basic patient characteristics did not differ between the two cohorts. Because only deceased patients were included in the analysis and most patients were admitted unplanned or as emergency admissions, generalizability of the results to a general ICU patient population should be avoided.

## 5. Conclusions

Taken together, we conclude that during a ten-year period from 2009 to 2019 the frequency of EOLDs and the completion rate of advance directives have increased considerably. In addition, EOLD communication with the patients´ next of kin and EOLD-associated documentation have further improved. Whether these changes also impact the EOLD process should be a subject of further research.

## Figures and Tables

**Table 1:** Patient characteristics.

	All (*n* = 229)		2009 (*n* = 123)		2019 (*n* = 106)		*p* Value
Age, years, mean (±SD)	69.45	(±14.47)	71.05	(±13.98)	67.59	(±14.88)	0.060
Gender, male, *n* (%)	140	(61.1)	77	(62.6)	63	(59.4)	0.624
Urgency of admission, *n* (%)							
Elective	52	(22.7)	31	(25.2)	21	(19.8)	0.063
Unplanned	44	(19.2)	29	(23.6)	15	(14.2)	
Emergency	133	(58.1)	63	(51.2)	70	(66.0)	
Severity Scores, mean (±SD)							
APACHE II	30.30	(8.36)	31.00	(8.06)	29.49	(8.66)	0.198
SAPS2	61.76	(17.73)	62.76	(17.19)	60.59	(18.34)	0.244
ICU-LOS, days, mean (±SD)	14.30	(24.01)	13.87	(20.00)	14.80	(28.05)	0.868

**Table 2:** Patient comorbidities.

Comorbidity. *n* (%)	All (*n* = 229)		2009 (*n* = 123)		2019 (*n* = 106)		*p* Value
Liver cirrhosis	18	(7.9)	10	(8.1)	8	(7.5)	0.870
Portal hypertension	10	(4.4)	6	(4.9)	4	(3.8)	0.755
Status post esophageal bleeding	8	(3.5)	4	(3.3)	4	(3.8)	1.000
Hepatic encephalopathy	3	(1.3)	2	(1.6)	1	(0.9)	1.000
Heart failure NYHA IV	35	(15.3)	26	(21.1)	9	(8.5)	0.008
Chronic pulmonary disease	52	(22.7)	29	(23.6)	23	(21.7)	0.735
Chronic obstructive pulmonary disease (COPD)	43	(18.8)	25	(20.3)	18	(17.0)	0.518
Lung fibrosis	5	(2.2)	1	(0.8)	4	(3.8)	0.185
Terminal renal insufficiency	26	(11.4)	10	(8.1)	16	(15.1)	0.098
Steroid medication	11	(4.8)	4	(3.3)	7	(6.6)	0.354
Chemotherapy	14	(6.1)	8	(6.5)	6	(5.7)	0.790
Immunosuppression therapy	8	(3.5)	3	(2.4)	5	(4.7)	0.476
HIV-Infection status positive	2	(0.9)	1	(0.8)	1	(0.9)	1.000
Leukemia	4	(1.7)	1	(0.8)	3	(2.8)	0.338
Lymphoma	4	(1.7)	3	(2.4)	1	(0.9)	0.626
Metastatic cancer	30	(13.1)	18	(14.6)	12	(11.3)	0.459

**Table 3:** Prevalence of EOLD and patient advance directives.

	2009 (*n* = 123)		2019 (*n* = 106)		*p* Value
EOLD, *n* (%)	87	(70.7)	91	(85.8)	0.006
Advance directive, *n* (%)	11	(8.9)	28	(26.4)	<0.001
Precautionary power of attorney, *n* (%)	10	(8.1)	32	(30.2)	<0.001
Legal guardian during ICU stay, *n* (%)	51	(41.5)	31	(29.2)	0.054
Documentation in PDMS special section, *n* (%)	23	(18.7)	53	(50.0)	<0.001

**Table 4:** Prevalence of advance directives and the EOLD process in patients deceased with EOLD.

	2009 (*n* = 87)		2019 (*n* = 91)		*p* Value
Advance directive, *n* (%)	11	(12.6)	27	(29.7)	0.006
Precautionary power of attorney, *n* (%)	9	(10.3)	31	(34.1)	<0.001
Legal guardian during ICU stay, *n* (%)	41	(47.1)	30	(33.0)	0.054
Documentation in PDMS special section, *n* (%)	23	(26.4)	50	(54.9)	<0.001
EOLD-Process, *n* (%)					
Patient participated in EOLD	7	(8.0)	7	(7.7)	0.930
Patient was informed of EOLD	7	(8.0)	7	(7.7)	0.930
Family/Surrogate decision maker participated in EOLD	53	(60.9)	68	(74.7)	0.048
Family/Surrogate decision maker was informed of EOLD	77	(88.5)	88	(96.7)	0.045
Successive decision from DNR to WH/WDLS	12	(13.8)	5	(5.5)	0.060
Multi-Step approach for WH/WDLS	27	(31.0)	34	(37.4)	0.374
Time ICU admission until EOLD, MW (±SD)	13.33	(±19.47)	13.60	(±28.48)	0.938
Time EOLD until death, MW (±SD)	3.48	(±6.40)	2.97	(±5.95)	0.278

## Data Availability

The datasets used and/or analyzed during the current study are available from the corresponding author on reasonable request.
